# Evaluation of Swab and Rinse Sampling Procedures and Recovery Rate Determination in Cleaning Validation Considering Various Surfaces, Amount and Nature of the Residues and Contaminants

**DOI:** 10.22037/ijpr.2020.1101173

**Published:** 2020

**Authors:** Somayeh Lamei Ramandi, Ramin Asgharian

**Affiliations:** a *Department of Analytical Chemistry, Faculty of Chemistry, Islamic Azad University, North Tehran Branch, Iran. *; b *Department of Pharmaceutics, Faculty of Pharmacy, Islamic Azad University of Pharmaceutical Sciences Branch, Tehran, Iran.*

**Keywords:** Cleaning validation, Recovery, Sampling, Swab, Rinse

## Abstract

A cleaning validation for a family of compounds utilizing swab sampling and rinse solution procedures, and high performance liquid chromatography for separation and detection of the analytes was performed.Effective parameters on recovery including sampling method, swab characteristics, solvent, swabbing technique, and material substance of product contact surfaces within the manufacturing equipment for swab and rinse sampling method, quantitative cleaning verification method, and active pharmaceutical ingredient (API) level and nature have been studied.The limit of detection and the limit of quantitation for the HPLC method were determined to be 0.0198 µg/mL, and 0.0495 µg/mL of the analyte, respectively. The linearity on replicate injections of the standard prepared in the range of 0.78, 1.55, 3.1, and 6.2 µg/mL, and relative standard deviation (R.S.D.) found to be 1.2, 1.0, 0.9, and 0.6, respectively with correlation coefficient of R^2^ = 0.9999. Recovery coverage for each type of surface was acceptable, ranging from 63.88% for swab sampling of stainless steel to 97.85% for rinse sampling of PVC. The acceptance criteria for precision on replicate injections of the analyte prepared in three concentration levels covering the specified range of 50, 100, and 200% was successfully accomplished R.S.D. lower than 15% for recovery results.Thus, choosing the appropriate sampling method, swab type, and surface condition can affect and increase recovery rate determination efficiency.

## Introduction

Validation of cleaning procedures has generated considerable discussion in pharmaceutical industry. A number of products have been recalled over the past decades due to cross-contamination and inadequate cleaning (2). Cleaning validation plays an important role in reducing the possibility of product contamination from pharmaceutical manufacturing equipment.

It demonstrates that the cleaning process adequately and consistently removes product residues, process residues, and environmental contaminants from the manufacturing equipment/system, so that this equipment/system can be safely used for the manufacture of specified subsequent products which may be the same or a different product.

Product may be a drug product, active pharmaceutical ingredient, intermediate, or another type of formulation (9). The cGMPs and U.S. Food and Drug Administration (FDA) have provided the pharmaceutical industry with general guidance for cleaning requirements.

Equipment and utensils shall be cleaned, maintained, and sanitized at appropriate intervals to prevent malfunctions or contamination that would alter the safety,

identity, strength, quality, or purity of the drug product beyond the official or other established requirements (2, 11). The cleaning validation involves a series of stages over the lifecycle of the product and cleaning process including cleaning process design, cleaning process qualification, and continued cleaning process verification. Cleaning process design intends to design, develop and understand the cleaning process residues and to establish the strategy for the cleaning process control. In cleaning process qualification, it should be demonstrated that the cleaning procedure works as expected for qualification of specific equipment used in the cleaning such as clean in place (CIP) systems, cleaning operational parameters (temperature, flow rates, pressure), identification of the most difficult cleaning locations, and training of operators. Continued cleaning process verification stage demonstrates that the cleaning process remains in control throughout the product lifecycle (8). The cleaning procedure should perform an appropriate number of times based on a risk assessment and meet the acceptance criteria in order to prove that the cleaning method is validated (1, 13, 14, 17). There are two general types of sampling that have been found acceptable. The most desirable is the direct method of sampling the surface of the equipment. Another method is the use of rinse solutions (2). Sampling materials and method should not influence the result. Recovery should be shown to be possible from all product contact materials sampled in the equipment with all the sampling methods used (1). Cleaning procedures, protocols and reports must be documented appropriately. Cleaning and use log should be established (12). In cleaning validation protocols the following items should be specified: sampling locations, the relevant selection rational, and acceptance criteria. (1). the advantage of direct sampling is the possibility of evaluating hardest to clean and accessible areas, leading to establishing a level of contamination or residue per given surface area. Additionally, residues that are “dried out” or are insoluble can be sampled by physical removal (2). The disadvantage of this sampling method for often complex API equipment is that difficult to reach areas may not be accessible by swabbing. Nevertheless, these areas may be the critical areas for the determination of the amount of residue in the equipment (8). In the cases where swabbing is not possible, for example restricted access, swabbing may be substituted by the analysis of final rinse solutions. Rinse samples can be used to determine the carryover of residues over a large surface area and cover all main process items including transfer pipework. In the cases where swab sampling is not practical, it is acceptable to analyse only rinse samples; however this should be justified as part of the validation study. Two advantages of using rinse samples are that a larger surface area may be sampled, and inaccessible systems or ones that cannot be routinely disassembled can be sampled and evaluated (2, 8, 18). Therefore, if appropriately designed, this method will give the best indication of the amount of residue remaining in the equipment. A disadvantage of rinse samples is that the residue or contaminant may not be soluble or may be physically occluded in the equipment (8). A sample isolated by sampling methods, should be analysed by a suitable analytical method e.g. total organic carbon (TOC) (10, 17), high performance liquid chromatography (HPLC), gas chromatography (GC), GC-MS, thin layer chromatography (TLC), dry residue, UV photometric, titration, conductivity or pH. The suitability of the method can be documented by appropriate validation (8-20).

## Experimental

*Material and reagents*

HPLC grade of methanol was obtained from Merck (Germany). Water was prepared freshly using a Puris Expe-UP water purification system (Korea). USP Chlordiazepoxide RS was used as reference standard.

*Swabs and surfaces*

Alpha swabs (Texwipe® 761) were from Texwipe (Philippines). Test surfaces including Stainless Steel, Polyvinyl Chloride (PVC), Poly (methyl methacrylate), also known as Plexiglas for swab sampling and Polyvinyl Chloride (PVC), and Polyester for rinse sampling were constructed in house with dimensions of 5cm×5cm. The coupons were representative of product contact surfaces within the manufacturing and packaging area. Prior, the selected surface areas were washed and ultrasonicated in water. After ultrasonication the surfaces were rinsed with purified water, and dried at room temperature.

*Instrument*

The experiments were performed on Waters Alliance series HPLC system, Empower software, auto sampler and pump model 2695, column compartment RT-85 °C, and UV-VIS detector model 2487 (USA). 

*Chromatographic condition*

The mobile phase was prepared by mixing 60% of HPLC grade of methanol and 40% of water. The mixture was degassed by sonication for 5 min. Operating conditions of HPLC utilized in this study were performed under isocratic elution. A Phenomenex C18 column with a particle size of 5 µm (250mm×4.6mm), 25 °C column temperature, at a flow rate of 1.0 mL/min, and 5 µL injection volume was used. Detection at UV 254 nm was applied. The retention time of Chlordiazepoxide was approximately 9 min. 

*Standard preparation*

A stock standard was prepared by weighing approximately 6.2 mg of USP Chlordiazepoxide RS into a 100 mL volumetric flask, and dissolving it in mobile phase. The standard solution was diluted from the stock solutions with Methanol and water (60:40) solvent mix (6.2 µg/mL).

*Sampling method*

In order to demonstrate that the plant equipment is verified clean and meets the pre-defined acceptance criteria, justification should be provided for the selection of the appropriate verification technique on a case by case basis. A combination of the swab and rinse sampling methods is generally the most desirable. Swab sampling of the direct surface is designed to test small sections of the equipment surface for the presence of residues. Samples should be taken from all main equipment items and since swab sampling does not cover the entire equipment surface area, justification should be provided for the choice of the area for swabbing. The swab sample can then be extracted and examined using a suitable analytical method. The quantified residue obtained from the sample is then extrapolated to the whole equipment (8). The type of sampling material used and its impact on the test data need to be determined since the sampling material may interfere with the test (2). Rinse sampling outlines the quantitation of the amount of residue remaining in the equipment after cleaning based on the amount of residue in the last rinse of the routinely used cleaning procedure. The residue amount in the equipment can be assumed to be equal to the amount of residue in the last wash or rinse solvent portion. The assumption is based on the worst case consideration that a further rinse (or any reaction) would not remove more than the same amount of residue present in the analysed rinse sample. For quantitation, a solvent sample is removed and the residue in the sample is determined by a suitable analytical method, which can then be extrapolated to the whole equipment (8). A direct measurement of the residue or contaminant should be made for the rinse water when it is used to validate the cleaning process. It is not acceptable to simply test rinse water for water quality rather than to test it for potential contaminates (2).

*Swab and rinse sample preparation*

Frames made of Polytetrafluoroethylene (PTFE) as chemically inert material with dimensions of 5cm×5cm were placed over the surfaces to be sampled. Spiked surfaces were prepared by adding spiking standard onto the model surfaces, and letting to dry at room temperature prior to swabbing. Two swabs were used subsequently. Purified water was used as the solvent to wet the first swab. The second swab was used dry. A swab sample was prepared by wiping horizontally on one side of the swab, flipping the swab and wiping vertically on the other side of the swab. Each swab sample was then placed in a test tube. Desorption of the swabs and extraction of the residues was done by adding Methanol and water (60:40) solvent mix and hand shaking for approximately 2 min. 

Rinse-sampling was performed with purified water. The aim was to make sure that the rinse sample is directly related to the remained target residue which was defined as the worst case and rinse procedure is appropriate to remove the residue from model surfaces validated in recovery studies. Spiking standard was pipetted from stock solution to the model surfaces. After drying at room temperature, Methanol and water (60:40) solvent mix was used to rinse the model sheet to a plate and shaking approximately 5 min on a shaker. The extract was transferred into a test tube.

*Establishing cleaning limits*

FDA does not set acceptance limits for the manufacturers. Specific analytical acceptance criteria for target residues must be established by the manufacturer based on a practical, achievable, and verifiable determination practice. It is important to define the sensitivity of the analytical methods in order to set reasonable limits (2). The starting point for any determination of residue acceptance limits is the amount of residue from the cleaning process that could be present in the subsequently manufactured product without posing an unreasonable risk (4) while the acceptance limit in the next product, of surface contamination, or of the analyzed sample is interrelated; they are not of the same units. In the contamination of the next product the units are ppm or µg/g, for surface contamination the units are µg/cm^2^, and for the analyzed sample the units are µg or µg/g. Limits per surface area are not comparable directly without batch size and equipment surface area. Although the Limits in the subsequent product are the same as limits in the analyzed sample, they also are not comparable without relevant information to area swabbed and the swab recovery factor. The FDA mentions limits proposed by industry representatives, such as 10 ppm, biological activity levels such as 0.1% of the normal therapeutic dose, and organoleptic levels such as no visible residue. The published Lilly criteria are that the equipment is visually clean, any active agent is present in a subsequently produced product at maximum levels of 10 ppm, and any active agent is present in a subsequently produced product at maximum levels of 0.1% of the minimum daily dose of the active agent in a maximum daily dose of the subsequent product.

$\mathrm{MAC}=\frac{\left(\mathrm{STD}\times \mathrm{BS}\times \mathrm{SF}\right)}{\mathrm{LDD}}$

MAC is the maximum allowable carryover, STD is the minimum daily dose of active in product A, BS is the smallest batch size of the subsequent product, SF is a safety factor (0.001), and LDD is the maximum daily dose of the subsequent product.

AL) is the acceptance limit for residues in µg/dm^2^. SA is the swabbed surface area, R is the recovery of the sampling method and TSA is the total surface area of production line in direct contact with the product (4).

$(\mathrm{AL})=\frac{\left(\mathrm{MAC}\times \mathrm{SA}\times R\right)}{\mathrm{TSA}}$

*The Worst case determination*

The worst case rating prioritizes existing drug substances in a cleaning validation program based on investigations and risk assessment on solubility, potency, the lowest therapeutic dose or toxicity data (LD50), the lowest acceptable daily exposure (ADE) or permitted daily exposure (PDE), cleanability, and dosage form to present documented evidence supporting the scientific rating for each criterion (1, 4, 8).

*Method validation*

The method was validated for specificity, limit of detection, limit of quantitation, precision, accuracy, and surface recovery, in accordance to recent references (5-6-19). For system suitability, five repetitions of injection from standard solution were used .The acceptance criteria for system suitability were as follows: column efficiency is not less than 3600 theoretical plates, tailing factor is not more than 2.0, and relative standard deviation is not more than 2.0% (5).

*Linearity*

**Figure 1:**
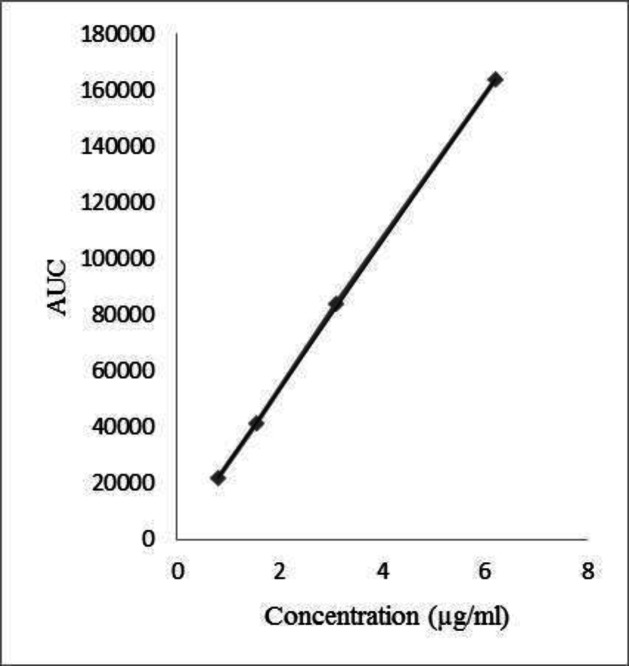
Calibration curve of Chlordiazepoxide

Calibration of the instrument was done to determine linearity of the method. Linearity was studied by analyzing a series of standard solutions containing 0.78, 1.55, 3.1, and 6.2 µg/mL Chlordiazepoxide by diluting stock standard solution in Methanol and water (60:40). Table 1 summarizes the results of the linearity study. Calibration curve of Chlordiazepoxide is shown in Figure 1.

*Accuracy*

**Figure 2:**
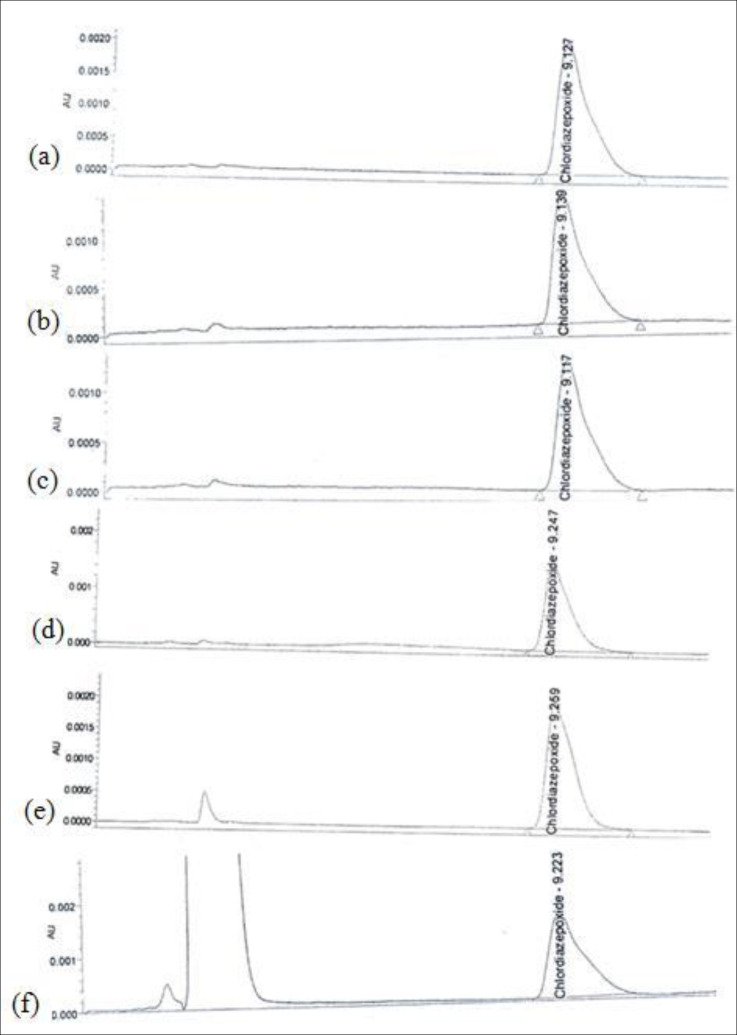
Chromatograms of Chlordiazepoxide recovery studies at 100% concentration level: (a) Standard solution; (b) Stainless Steel swab sampling; (c) PVC swab sampling; (d) Plexiglas swab sampling; (e) PVC rinse sampling; (f) Polyester rinse sampling

The accuracy of an analytical procedure is the closeness of test results obtained by that procedure to the true value and it should be established across its range. Accuracy is calculated as the percentage of recovery by the assay of the known added amount of analyte in the sample, or as the difference between the mean and the accepted true value, together with confidence intervals (5). Both swab and rinse sample concentrations were determined by reference to calibration line. Nine determinations of reference material in three concentration levels covering the specified range including 50, 100, 200%, and three replicates of each concentration were spiked to 5cm×5cm model surfaces. Figure 2 represents chromatograms of recovery studies at 100% concentration level from pre-defined surfaces. There should be evidenced that samples are accurately recovered. A recovery of >80% is considered good, >50% reasonable and <50% questionable (7). Table 2 indicates acceptable accuracy for recovery studies in three concentration levels for swab and rinse sampling from different types of surfaces.

*Precision*

Precision is the degree of agreement among individual results. It should be measured by the scatter of individual results from the mean and expressed as the relative standard deviation (R.S.D.) (7). Precision of the method was examined by preparing and analyzing spiked replicates and relative standard deviation of recovery data in three concentrations for each type of surface. As shown in Table 3, the acceptance criteria for precision, R.S.D. lower than 15% was successfully accomplished.

*LOD and LOQ*

The LOD and LOQ of the method were found to be 0.0198 µg/mL and 0.0495 µg/mL, respectively based on the analytical method validation of chlordiazepoxide assay.

*Specificity*

No interference from common excipients in the formulation was observed.

## Results and Discussion

In order to increase the recovery of spiked pre-defined product contact surfaces, two swabs were used. Water was used as the solvent for wetting the swabs. The second swab was used wet and dry for stainless steel surfaces. The recovery percentage of the surfaces which were swabbed by two swabs moisturized with water was lower than 50%. In the validation method the second swab was used dry to mop up the solvent residues left.

Various types of surface materials are in use in the production line. In order to adequately swab these potential surfaces and recover the analyte that could potentially remain on the surfaces after cleaning, the accuracy of the method was investigated on all surface materials. During this validation, the worst-case recovery was utilized to establish recovery coverage percentage. For example, recovery coverage percentage for stainless steel surfaces was found to be 63.88%, and 70.68% at 200%, and 50% concentration levels, respectively. The recovery of 63.88% was utilized as the recovery coverage for all stainless steel concentration levels when the swab method was executed. 

When the recovery of the material substance is lower than 50% or it is hard to clean area, it can be dedicated to the product.

Failure mode and effect analysis (FMEA) risk assessment was done for cleaning validation. In order to mitigate the risk of inappropriate cleaning of hard to clean areas due to operator mistake, both swab and rinse samples were taken from blistering machine feeder brush as hardest to clean area in validation of three consecutive batches. Although the results were within the limit, the feeder brush was considered as dedicated to each product to eliminate product defect by preventing, correcting, or drawing attention to human errors.

**Table 1:** Statistical data of calibration curve of Assay

**Analyte (Chlordiazepoxide**)	
Regression equation	Y = 26212x+1624.7
Linearity range	0.78-6.2 (µg/mL)
y-intercept	1624.7
Slope	26212
Correlation coefficient	0.9999

**Table 2:** Accuracy of the method for material substance of product contact surfaces in three concentration levels

**Material substance **	**Sampling Method**	**Concentration Levels**		
		50%	100%	200%
Stainless Steel	Swab	70.68	67.18	63.88
Polyvinyl Chloride (PVC)	Swab	69.36	66.71	78.06
Poly(methyl methacrylate) Plexiglas	Swab	92.90	84.51	77.10
Polyvinyl Chloride (PVC)	Rinse	96.74	96.52	97.85
Polyester	Rinse	82.61	74.90	71.98

**Table 3:** Precision of the method for material substance of product contact surfaces in three concentration levels

**Material substance **	**Sampling Method**	**Concentration Levels**		
		50%	100%	200%
Stainless Steel	Swab	1.7	8.1	7.1
Polyvinyl Chloride (PVC)	Swab	4.9	11.4	4.9
Poly(methyl methacrylate) Plexiglas	Swab	1.0	13.5	3.9
Polyvinyl Chloride (PVC)	Rinse	3.6	1.6	4.0
Polyester	Rinse	1.8	10.1	12.6

## Conclusion

The purpose of this study was to develop a method for cleaning validation including worst case determination, swab and rinse sampling, recovery studies, manufacturing and packaging at pre-defined product contact surfaces. The method is specific, linear, accurate, and precise over defined concentration range.
